# Decision-Making Process in Comprehensive Medication Management Services: From the Understanding to the Development of a Theoretical Model

**DOI:** 10.3390/pharmacy8040180

**Published:** 2020-10-03

**Authors:** Isabela Viana Oliveira, Yone de Almeida Nascimento, Djenane Ramalho-de-Oliveira

**Affiliations:** 1 College of Pharmacy, Center for Pharmaceutical Care Studies, Federal University of Minas Gerais, Belo Horizonte 31270-901, Brazil; djenane.oliveira@gmail.com; 2 Centro Universitário Newton Paiva, Belo Horizonte 30431-189, Brazil; yone.almeida1@gmail.com

**Keywords:** comprehensive medication management, decision-making process, grounded theory, pharmaceutical care

## Abstract

In Comprehensive medication management (CMM), the practitioner applies a decision-making method to assess patients’ pharmacotherapy in order to identify and solve drug therapy problems. Grounded theory was used to understand how pharmacists make clinical decisions when providing CMM service. Data collection included individual semi structured interviews with 11 pharmacists, observation of clinical case discussions and CMM consultations provided by the participating pharmacists. Two main categories emerged: 1. Understanding the rational method of decision-making: the foundation of the patient care process. 2. Balancing the care equation: the objective and the subjective, which includes a theoretical proposal explaining the pharmacists’ decision-making process and the factors that can modify this process. The pharmacotherapy knowledge should guide the anamnesis. Thus, the professional can evaluate the indication, effectiveness, safety and convenience of medications used by the patient. After exploring patients’ medication experiences, pharmacists can follow two courses of action: helping the patient overcome barriers to medication use; or matching the pharmacotherapy to the patient’s routine. Professional autonomy and absence of the patient at the time of the decision were some factors that influenced the pharmacist’s decision. Findings provide a broad understanding of pharmacists’ decision-making process during the care of patients using medications. It can be applied as a basis for educational interventions to train professionals on decision-making.

## 1. Introduction

Comprehensive medication management (CMM) is a term used to designate a standard of care in which all medications used by a patient are evaluated individually to ensure that each of them is appropriate, effective for a particular health problem, safe considering all the comorbidities and other medications in use, and able to be taken by the patient as recommended [1]. This service is based on the theoretical framework proposed by pharmaceutical care practice, which is a professional practice that defines the philosophy, the patient care process and a management system for the clinical pharmacist [2]. The practitioner provides the CMM service to optimize medication use and improve the clinical outcomes of patients through the management of drug-therapy in a patient-centered manner. Previous research has demonstrated the clinical and economic impact of this intervention across multiple settings and patient populations [3,4,5,6,7,8].

The standard of care established by CMM implies using the rational method of decision-making on pharmacotherapy proposed by pharmaceutical care practice, in which all medications used by a patient are evaluated according to their indication, effectiveness, safety and convenience for that specific patient and in that particular order. Clinical pharmacists use this logical process to identify and solve drug-therapy problems (DTP), thus helping patients to achieve the best results from their medications [2]. As stated by the document recently published by The American College of Clinical Pharmacy (ACCP) [9], the order proposed for the medication assessment is intentional as “one must determine whether an indication is correct for a medication before effectiveness, safety, and adherence are considered. Ensuring adherence is a final step in the assessment.”

This rational method of decision-making on the pharmacotherapy plays a fundamental role in the organization of professional thinking when caring for patients, as well as in keeping pharmacists focused on their major professional responsibilities [10]. However, there are still few studies focusing on the full decision-making process of the provider of CMM services [11]. Other professions with established clinical practices, such as nursing and medicine, have studied their decision-making process extensively [12,13,14,15,16,17,18,19].

The clinical decision-making process involves skills such as critical thinking and problem solving [20]. It is a process that includes everything from clinical reasoning to decision-making. According to Simmons [21], clinical reasoning is a complex cognitive process that uses formal and informal thinking strategies to gather and analyze patient information, evaluate the importance of this information, and weigh alternative actions. Thus, the rational method used during the provision of CMM can be understood as clinical reasoning in pharmacotherapy applied by the clinical pharmacist when caring for patients. Decision-making, in turn, is defined as the outcome of the thought process [22].

Recent studies have pointed out CMM as the practice standard that should be adopted by pharmacists in patient care [1,9,23,24,25]. It is, therefore, an expanding service, and the understanding of the clinical decision-making process, based on the experience of professionals who are in practice, can contribute to the development of strategies for adequate training of students and pharmacists for this service.

Thus, considering the importance of incorporating pedagogical innovations to improve clinical education [26], this study aimed to construct a theoretical model for the decision-making process during the provision of CMM services. The question that guided this research was: how does the pharmacist’s decision-making process take place during the provision of CMM services? This process includes the rational decision-making method described here and how the pharmacist has incorporated it; but it goes further, encompassing the interpersonal relationships that affect the way decisions are made in the real world.

## 2. Materials and Methods

The methodology used in this study was grounded theory, as proposed by Kathy Charmaz [27]. According to this author, “grounded theory is constructed through our involvements and interactions with people, perspectives, and research practices, […] offering an interpretive picture of the studied world [27].” Thus, the methodological approach proposed by Charmaz [27] is aligned with the positioning that we assume as researchers since we believe that there is no separation between the researcher and the researched but rather that knowledge is constructed from the processing of meanings in the researcher’s mind [28].

The decision-making process includes establishing social relationships and involves some abstract concepts that need to be connected to be explained. The grounded theory allows one to understand how this process takes place and construct a theoretical model that, as emphasized by Strauss and Corbin [29], provides a meaningful guide to action. Therefore, this methodology allowed the construction of a model that could become a guide for the decision-making process of the CMM provider. In addition, our purpose was not to use existing decision-making theories to understand the decision-making process of the pharmacist during the offering of CMM, but to be open for the emerging data to show the way to a new understanding of this process.

### 2.1. Participants and Data Collection

The participants in this study were pharmacists that provide CMM services in different practice settings of the following Brazilian cities: Belo Horizonte, Contagem, Lagoa Santa, Uberlândia, Salvador e Ribeirão Preto. Eleven participants who routinely perform the clinical decision-making process during the provision of CMM services were selected, thus being key informants to understand this process. They consisted of 5 pharmacists practicing in primary care clinics, 3 in specialty clinics, in both private and public health systems: 2 in clinics at universities and one in a public pharmacy. Data collection occurred between November 2014 and December 2015.

In grounded theory, the sample is not previously defined. Thus, data collection began with the interview of one pharmacist with more than five years of experience with the provision of CMM services. From the analysis of this first interview, new data were collected based on the themes that emerged. The selection of the following participants was also made according to the new insights gained from subsequent data analysis. In this process, priority was given to include pharmacists from diverse practice scenarios and level of experience with CMM services, for a richer comparison between the data. New participants were added until theoretical saturation was reached.

Semi-structured interviews (Appendix A) were conducted with eleven pharmacists. All interviews were audio-recorded and transcribed verbatim for data analysis. The interviews had an average duration of 71 min, ranging from 45 to 94 min.

In addition, data were also collected from observation of CMM consultations provided by seven participant pharmacists. During the thirteen months of data collection, the patient/ pharmacists’ interactions were observed in some locations where participants provided CMM services. In general, observations of CMM consultations preceded the interviews. At the end of the research, fourteen consultations were observed.

Case discussions of patients attended by participating pharmacists were also the subject of field observations. Thus, data were also collected during observations of patient case discussions at meetings of the Pharmaceutical Care Group, a group that has met every two weeks since 2003 to discuss patient cases followed up by CMM pharmacists of Belo Horizonte, Brazil. Discussions of seven clinical cases were observed.

In grounded theory, the observation prioritizes the studied process rather than the research environment itself [27]. Therefore, when observing the CMM consultations and discussions of patient cases between pharmacists or between pharmacists and students, the researchers turned their gaze to the decision-making process, recording in a field diary everything relevant to the understanding of this phenomenon.

This study was approved by the Ethics Committee of the Federal University of Minas Gerais (UFMG) (process number CAAE-25780314.4.0000.5149). All participants provide signed informed consent.

### 2.2. Data Analysis

Data analysis occurred simultaneously to the data collection. The first interview was transcribed and then its coding was performed line-by-line, i.e., each line of the transcript was named according to its meaning. This first analysis directed the subsequent data collection, and this process was carried out throughout the research, always comparing the data within the same interview, between interviews and between interviews and observations.

Focused coding, in which the most frequent and significant initial codes were used to integrate and organize large amounts of data, started with the evolution of the analysis. Data analysis is a dynamic process, and during this stage, it was sometimes necessary to resume the line-by-line coding for new collected data. According to the development of the analysis, some initial codes were renamed to fit the data, and at the same time they reached a higher level of abstraction. The theoretical sampling, when guiding the research progress, directing the search for more data that explained better categories, allowed the development of refined and solid categories [27].

In addition, memos were written with the purpose of comparing the data, exploring the ideas about the codes and directing the new data collection throughout the analytical process. The memos had explanations about the characteristics of codes and the relations between them and the literature. The writing of memos was a way of recording all the way traveled by the researcher until the construction of the theoretical model.

## 3. Results and Discussion

The participants were named as P1, P2, etc. The characteristics of each pharmacist are reported on Table 1.

**Figure 1:**
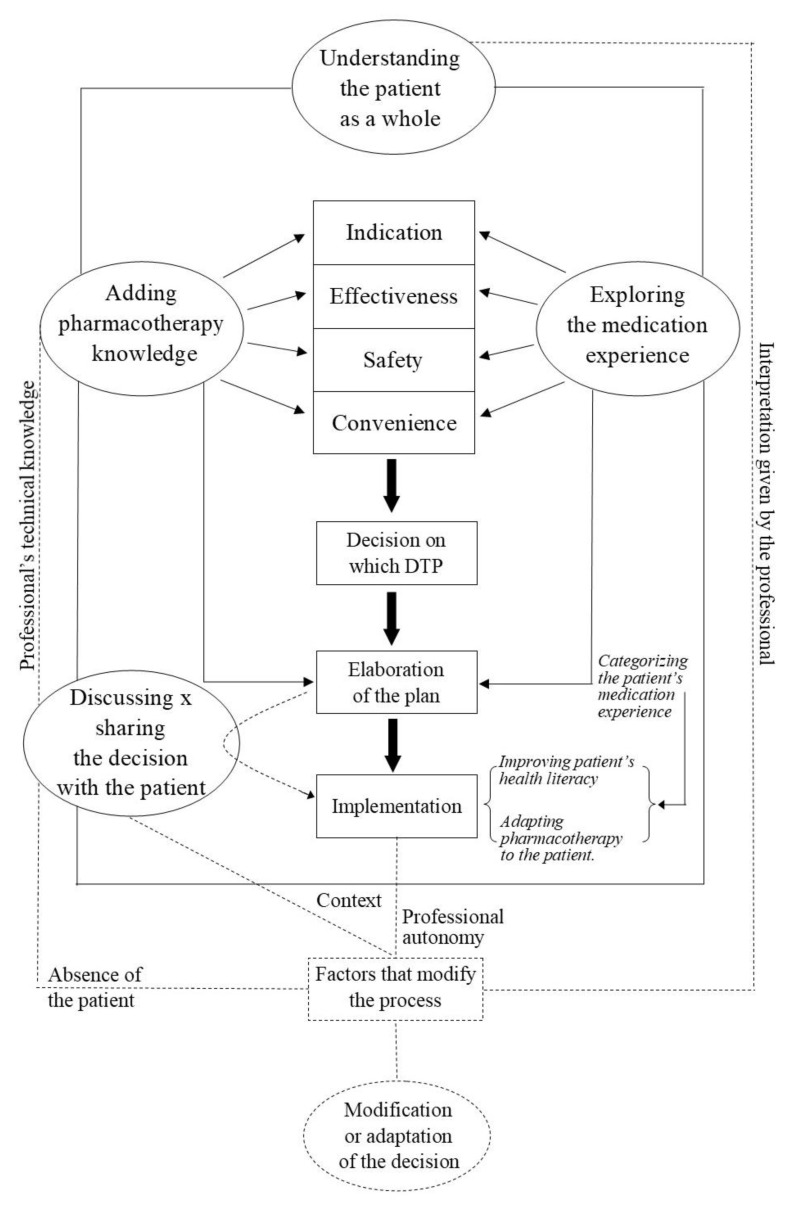
Model proposal for the pharmacist’s decision-making process.

After a systematic analysis of the data, two main categories were constructed. The first one was named “Understanding the rational method of decision-making: the foundation of the patient care process.” This category reveals the meaning that the clinical reasoning in pharmacotherapy proposed within the framework of pharmaceutical care practice acquires for pharmacists who are in practice. The second category, “Balancing the care equation: the objective and the subjective,” was divided into the following four subcategories: “Adding pharmacotherapy knowledge”; “Exploring the patient’s medication experience”; “Discussing versus sharing the decision with the patient: what are we really doing?” and “Factors that can modify the decision-making process”. This second category and its subcategories translates how pharmacists make decisions during the provision of CMM services, giving rise to the proposal of a theoretical structure for this decision-making process (Figure 1).

### 3.1. Understanding the Rational Method of Decision-Making: The Foundation of the Patient Care Process

During the provision of CMM services, the assessment of patients’ medications follows the order of indication, effectiveness, safety and, finally, the convenience to the patient. This rational method for making decisions in pharmacotherapy is at the origin of the decision-making process of the pharmacist who cares for patients in a person-centered way. Therefore, this category is the first to be presented because it deals with the structural basis of the developed theoretical model.

The rational method of decision-making in pharmacotherapy provides tranquility to the pharmacist, who is aware that his way of thinking and making decisions about medications will not vary from patient to patient:

“*I can provide the same care, the same quality of care to any patient regardless of the number of medications, or the number of health problems he/she has. Nowadays, I feel calmer… because it doesn’t matter which person is in front of me, I know what I am going to do with him/her. After a year of practice, I am already reaching 200 patients, and I can be relaxed because, with just a piece of paper in hand, I know what I have to do. That thanks to the method.*”

*P10*

It should be emphasized that using this structural basis with all patients does not mean ignoring their individual characteristics and context, which will be discussed later. The decision-making process makes the practice universal and reproducible while the patient remains unique. As pointed out by participants, peace of mind lies in knowing exactly how to assess a patient. In fact, the decision-making process supports the entire patient care process and not only the categorization of a patient’s drug therapy problems:

“*At all times, the patient arrives, shows me the prescription, and I am thinking like that. I look at each medication, is it indicated, effective, safe, convenient? It is the process in my head all the time. I cannot evaluate medications if it is not this way.*”

*P2*

“*When I am with the patient, with the medical record, with the literature, with the health team, I always have that sequence in my head, looking for ways to find all the answers.*”

*P1*

Having this sequence in mind at all times is essential for CMM pharmacists to be able to assess all relevant information (objective and subjective) to understand the pharmacotherapeutic needs of the patient, facilitating the identification and resolution of DTP. Ramalho-de-Oliveira [30] during her ethnography to understand the culture of pharmaceutical care practice in Northern United States, questioned one of the participating pharmacists on how she had reached the decision that a particular patient had a specific drug therapy. In agreement with the participants in this study, the pharmacist said she used a certain order of reasoning during the conversation with the patient, which directed her questions to cover these four points (indicated, effective, safe and convenient) for each and all medications [30].

In teaching the practice, it is necessary to stimulate students to use this thought process as a guide to their actions in collecting data, discussing cases with other professionals, searching the literature, and not only to identify a DTP. With regards to the importance of teaching the thought process to the pharmacy student since the first stages of their training, the participant P1 asserts: “It took me a long time to make these connections, but then when I realized it is the crux of the practice, I made it a priority in my teaching.” Therefore, if teaching does not favor such an association, practice learning is hindered, delaying the success of this new professional to manage patients’ drug therapy.

Participants emphasized that by insisting on clinical reasoning, the pharmacist can more clearly perceive the patient’s real DTP and the best way to solve it. In this sense, Freitas [31] underlines that in evaluating the medications used by the patient in relation to the indication, effectiveness, safety, and convenience, the pharmacist makes complete clinical judgments, without “forgetting anything,” which contributes to the quality of the provided care. Having this structural base allows the practice to be dynamic and, at the same time, the pharmacist becomes self-assured of his/her conduct. Notably, the clinical experience of the pharmacist and his/her prior knowledge remain fundamental; however, it is supported by the method:

“*Before I was surer about the decisions [the interviewee makes a sign of quotation marks] that I took, not a decision, but the follow-ups I made. For me that was good, and I looked at a patient’s prescription and already knew if it was good or not, but based on what? On posology, on drug interactions. Sometimes I saw a problem with the prescription, which was not a real problem for the patient, but I saw a problem. Or I did not see a problem in the prescription when the patient had a lot of real problems with his medications. However, I was sure at that time! I thought I was a good pharmacist. I knew the reasons all the medications were used… Then I started working with CMM, and I started to understand, to execute the reasoning [rational thought-process] more naturally, without having to be focusing only on the prescription. I overcame the phase of insecurity. I returned to certainty, but a more reasoned certainty. CMM became an architectural project in which my knowledge came back to fit into certain places, places that I could access in a more useful way.*”

*P7*

According to this participant, before performing the CMM service and, therefore, before adopting the rational method for decision-making on pharmacotherapy, she analyzed the prescription of the patient in relation to the dosage of medications and the presence or not of drug interactions. This analysis does not require critical thinking strategies, considering that it is only necessary to memorize doses and frequencies of medications under ideal conditions [31] and to use a software that informs the present interactions. According to Facione and Facione [32], critical thinking can be defined as “the process we use to make a judgment about what we believe and what to do about the symptoms that our patients present for diagnosis or treatment.” The solid use of these skills is essential for excellence in professional judgment [32]. Therefore, when caring for patients who use medications, critical thinking is imperative. Freitas and Ramalho-de-Oliveira [10] pointed out that the use of the method discussed here is strongly related to the application of critical thinking skills in pharmacy practice with a focus on the patient. The excerpt from the interview mentioned above suggests that without a guide for the evaluation of pharmacotherapy, the pharmacist does not develop strategies of critical thinking, and as the participant herself emphasizes, does not make rational decisions.

Another point that calls attention in the narrative of the pharmacist P7 is the identification of prescription problems that were not real for the patient and the recognition that real problems went unnoticed before using the thought-process. It also suggests that by understanding and applying the clinical reasoning, its use allows the pharmacist to reach the real patient and the world of that patient. It happens because the focus of the practice is on the problems associated with the use of medications by a real patient and not a problem related to a prescription independently of the patient. Thus, this reasoning is tied to the philosophy of practice embodied in CMM services and, therefore, in the understanding that the patient is at the center of care [8]. Thus, within CMM, the use of the rational decision-making process allows that the identification of DTP becomes contextualized in reality and that the pharmacist begins to identify and prioritize problems that are actually lived by the patient.

In the absence of the decision-making method to assess the patient’s pharmacotherapy, the pharmacist is floating, hovering over all the health needs of the patient, without recognizing what should be his/her major focus on patient care [33], and possibly acting only as a follower of decisions of other professionals. The participant P1 says “the decision-making method is the axis. I think when you do not understand it, practice makes no sense at all… it [method] makes very clear what your role is, and it does not let you escape from that.” The method is the foundation of the decision-making process and provides the organization of thought, encompassing all four dimensions (indication, effectiveness, safety and convenience) that must be evaluated when someone is using a medication. As well argued by a pharmacy student, these four dimensions allow the professional to be creative and propose solutions to the problem and, at the same time, let him/her to identify the boundaries of the practice and recognize how safe it will be to proceed with the management of drug-therapy [10]. It is what the pharmacist P7 calls “more reasoned security,” as the professional has a basis that supports him/her.

### 3.2. Balancing the Care Equation: The Objective and the Subjective

This category presents all the events that, when combined, make the pharmacist’s decision-making process complete, seeking to maintain the sequence in which they happen. The previous category is related to this one, and its understanding is important because the care process starts at the first contact with the patient and is anchored in the rational decision-making on pharmacotherapy. From this point, all participants demonstrated that they sought to combine technical objectivity with the subjectivity of the attended person in an attempt to balance this equation to identify the problem experienced by the patient and define how to solve it:

“*I say CMM is beautiful, it is an art, you take the technical piece and combine it with that wonderful thing that is the person, what he/she has, his/her beliefs, and experiences. Doing this is an art. I think it is incredible because it is not easy… When you get it, it is wonderful because the person realizes that you are taking that into account.*”

*P10*

The steps that make up the decision-making process during the offering of CMM services are presented in detail below.

#### 3.2.1. Adding Pharmacotherapy Knowledge

The technical knowledge used by pharmacists during the decision-making process is represented here by the knowledge of pharmacotherapy because it refers to how medications are used to treat and prevent diseases, thus involving the knowledge of the physiopathology of diseases and pharmacology. For a professional whose mission is to identify, prevent and solve drug-therapy problems, this knowledge is indispensable. According to Losinski [34], clinical knowledge, i.e., the knowledge about medications and health conditions, is the pillar that allows pharmacists to practice pharmaceutical care, necessary for patients and the healthcare team.

In this sense, this subcategory reveals how the pharmacotherapy knowledge is applied in the decision-making process of the professional during the provision of CMM service. This knowledge is needed from the first evaluation of the patient. One of the participants emphasizes the importance of solid training in pharmacotherapy so that during patient care the professional can direct their questions and associate this knowledge with clinical reasoning:

“*That is why I say that it is important to train the professional because he/she needs to know about pharmacotherapy to ask questions that are directed, to try to identify problems of effectiveness or safety.… the patient uses a calcium channel blocker, so I ask, ‘Do you feel anything?’ ‘No.’ ‘And edema? Does your foot swell? And then he/she says, ‘Look, my foot swells.’ ‘Oh, do you use diuretics? Do you have a cramp?’ All the time, I will ask questions to help me with this reasoning.*”

*P2*

Just as the method should guide the pharmacist during the evaluation of the patient, knowing pharmacotherapy will help the professional to be more focused. According to the medications used by the patients and their health conditions, the professional will know what questions he/she needs to ask to assess the indication, effectiveness, safety and convenience of medications in use by the person attended:

“*Understanding treatment protocols to know the indication, understand the disease, the parameters to monitor the effectiveness of the medication, and the safety profile of each medication to evaluate the safety. It is what will guide you. You have to think about the unique contribution you bring to care. It is your gaze. You have to combine the information to find specific problems there.*”

*P1*

Initiatives to integrate disciplines such as semiology and pharmacology have been carried out in Brazilian medical education, aiming at improving the clinical thinking abilities of these students. Contextualizing the teaching of pharmacotherapy with the rational method of decision-making and with disciplines that favor the application of this knowledge in patients’ assessment, such as semiology, may be a crucial strategy in the preparation of clinical pharmacists [35].

The following excerpt, taken from the field diary, shows how the pharmacists and student suspect a problem of effectiveness and raise hypotheses about its cause:

“*The patient had a request for a lab to evaluate the serum concentration of phenytoin and phenobarbital. Both student and pharmacist decided to wait for the test result to decide if it would be possible to increase the dose or to change to a more effective medication for that patient.*”

*(Excerpt from the field diary)*

From the results of the serum concentration, they will be able to confirm if the medications are within the recommended therapeutic range, indicating that the patient may need to change the treatment, or if the medications are below the expected concentration, suggesting the need to increase the dose. In this example, it is possible to observe the use of the hypothetical-deductive method, in which hypotheses are raised, and from the search for more information, they will be accepted or refuted [13]. Associated with the application of this method is the use of knowledge on the characteristics of the medications and their evaluation from the perspective of their indication, followed by the effectiveness, safety and, finally, convenience.

The importance of pharmacotherapeutic knowledge at the time of thinking about possible alternatives to solve a patient’s problem is highlighted by P7:

“*Looking for scientific studies, I was at this stage… there are good signs that atenolol can be a good option for the elderly. I said well, so that will be it, she needs another antihypertensive since she is in the maximum dose of losartan and hydrochlorothiazide… She used them correctly. I could not increase the dose of losartan nor hydrochlorothiazide. She needed another antihypertensive, and the natural thing was to add a beta-blocker that could also treat the migraine.*”

*P7*

Evidence-based pharmacotherapy is an approach in which the clinician evaluates the scientific evidence and its strength to support a therapeutic decision [36]. This pharmacist uses this strategy to make a decision and exemplifies how the scientific evidence was associated with the individual characteristics of the patient, who, in addition to reducing and ameliorating migraine attacks, required blood pressure control.

The results discussed so far indicate that it is not possible to manage drug therapy without knowledge of pharmacotherapy. This type of knowledge and the application of the rational approach to drug therapy go hand in hand and are essential in the decision-making process of the pharmacist who accepts the mission of caring for patients to identify, prevent and solve drug-therapy problems.

#### 3.2.2. Exploring the Patient’s Medication Experience

In the midst of the objectivity of scientific knowledge, all pharmacists have shown a disposition to incorporate the medication experience in managing patients’ drug therapy.

Pharmacists revealed to consider the patient’s experience not only to understand what it means to use medications on a daily basis but as a support for identifying the DTP experienced by the patient. The following excerpt from the field diary shows a participants’ discussion of a clinical case drawing attention to the possibility of the patient’s medication experience to signal a problem related to the need for medication:

“*The doubt was regarding a patient with a prescription of nortriptyline for depression, but who reported not taking it, as she claims to have no depression. For the student, who participated in the discussion, the patient had a problem with adherence to the treatment. However, it was argued the need to investigate whether the patient had depression to determine if there was an indication for the medication.*”

*(Excerpt from the field diary)*

In this case, the patient’s claim that she does not have depression announces an experience that must be explored to confirm if she really needs an antidepressant. Therefore, the pharmacist realized the need to seek more information to evaluate the need for this medication, working in collaboration with the physician, other professionals, and involving the patient. In this example, the association between the patient’s medication experience and the assessment of the pharmacotherapy (indication, effectiveness, safety and convenience) preserved the pharmacist to promote the use of a medication that might not be appropriate for the patient, demonstrating indisputably the importance of the method as a structural basis for the decision-making process.

Another participant describes how the medication experience, often expressed as an understanding and concern regarding pharmacotherapy, may direct the pharmacist to identify a drug therapy problem:

“*Regarding the patient’s concerns, he [patient] will tell me a lot about adverse reactions that are already happening or that he is afraid of because he heard about it or because he is not sure that it has a relationship with his medication, but it seems to have it, so it is a concern. The understanding is very much related to the behavior of the patient… So I think that it will inform much of the question of convenience.*”

*P11*

After deciding what problem the patient is experiencing, the pharmacist also considers the medication experience as a basis for professional intervention during the care process. Faced with the understanding of this experience, the professional tends to categorize such experiences because they can come from real perceptions or misconceptions:

“*I try to understand his experience, and I try to see where he is wrong or right.*”

*P3*

“*We clarify if those concerns are real or if they are unfounded. If they are real, how we will monitor them during the process… If the expectation is not correct, we work on it.*”

*P5*

It is important to emphasize that this strategy has nothing to do with the judgment of the attended person. On the contrary, the professional understands that he/she must consider the patient’s experience presented in order to contextualize the resolution of a problem to the specific situation he/she experiences. This categorization will indicate which path the professional should take with the patient. When understanding the patient’s experience, the pharmacist may perceive the need to make a different decision from the one previously planned. Alternatively, the professional decides that the patient’s experience needs to be explored so the patient can understand better her treatment and overcome the challenges of using it.

In certain situations, pharmacists have demonstrated how the medication experience can lead to a change in the professional’s behavior so that the patient continues to achieve positive health results:

“*A hypertensive patient was taking an ACE inhibitor [angiotensin-converting enzyme]. It was indicated, effective, safe, and convenient medication in my evaluation. However, her mother had died from drug hepatitis… and the doctor said the suspected drug was an ACE inhibitor. The ACE inhibitor controlled the patient’s blood pressure… it was indicated, effective, apparently safe, but there was this concern. I could not tell her that she was not going to have drug-induced hepatitis, do you understand? The fact that her mother had hepatitis did not mean she would have it, but I also could not affirm that she would not have it. Thus, that fear interfered with the process of adherence of this patient. She was taking it, but it would come a moment, in my evaluation, that she would stop taking it. So why can’t we suggest the change this medication, get in touch with her doctor and ask for the change? I could have said, ‘Keep taking it, it will not happen to you, this medicine is great, you are not even sure if it was, in fact, the ACE inhibitor that caused your mom’s hepatitis.’ However, out of respect for the patient, we have constructed another therapeutic plan. We made a letter proposing the class change of the antihypertensive drug.*”

*P5*

In the pharmacist evaluation, the medication used by the patient to control hypertension was indicated, effective, safe and convenient. However, faced with the patient experience with the antihypertensive based on real perceptions, the professional did not hesitate to change her decision to avoid a possible loss of blood pressure control in the future.

On the other hand, pharmacists have shown that the medication experience is used as a subsidy to improve patient’s health literacy, a concept that encompasses “the degree to which individuals have the capacity to obtain, process, and understand basic health information and services needed to make appropriate health decisions [37].” Exploring this experience allows the professional to know the patient status and use strategies that facilitate the understanding of patients about their health problems and medications:

“*I have already seen a patient like this: ‘What do you think about insulin?’ ‘Horrible, those who use insulin die. My brother died because he started using insulin. Then I go to explore the story of his brother. ‘But did your brother control diabetes?’ ‘No, he ate sweets.’ Then I say, ‘So did he die because he used insulin or because his diabetes was out of control? Then the patient says, ‘Oh, it is because he did not control it, right?’ ‘Probably.’ Then when I see that he understood it, I start saying that insulin is the best option.*”

*P3*

In this narrative, the pharmacist briefly demonstrates how she identified that the patient had misconceptions and how she conducted the conversation to help him to understand that the medication might not have been responsible for his brother’s death. Thus, the patient’s understanding of the importance of insulin in his treatment may help him to overcome the challenges of using this medication.

#### 3.2.3. Discussing Versus Sharing the Decision with the Patient: What Are We Really Doing?

After identifying the problem experienced by the patient, combining technical knowledge with the patient’s experience of using medications in his/her daily life, the decision-making process of the pharmacist continues, seeking to combine these two forms of knowledge to finally decide what intervention will be implemented to solve the problem. At this stage, pharmacist and patient interact at varying degrees to make a decision.

According to the literature, shared decision-making begins at the moment that the professional explains to the attended person the need to consider the available alternatives as a team. Then, these alternatives should be described in detail, presenting the pros and cons of each option. After providing sufficient information to the patient, the professional should help him to explore these alternatives, form his preference, and thus decide what will be best [38,39]. In the analysis of decisions made during the observation of CMM consultations and those pointed out in the interviews, in general, the pharmacists are genuinely interested in knowing the patient’s opinion about a decision:

“*The order I follow is: I identify the DTP, I share my choice to resolve the DTP with the patient because all my interventions are agreed upon with him. I always ask, ‘Can we do it? Can it be this way?’ He says, ‘It may be this way,’ and then I go to the prescriber and talk to him.*”

*P2*

In these excerpts, pharmacists develop a partnership with patients to discuss their decision, emphasizing the need for an agreement. Therefore, patients influence pharmacists’ decision-making process. They also play an important role in agreeing or not with the plan being developed. The experience of patients with CMM service reveals that they feel welcomed and in control of their health when they are asked if they agree with a certain choice or when they discuss their treatments and can clarify their doubts [40,41]. However, the degree of the patient involvement when participating in a discussion about the decision is not enough to configure it as shared-decision making, as there was no description of the available alternatives nor a joint decision about the best option. Towle et al. [42] point out that it is unlikely that a decision can be shared without offering options to the patient. A balanced situation is necessary, in which the alternatives presented and discussed with the patient are viable options and, therefore, the decision can be shared, justifying the use of this term [43].

Effectively incorporating shared decision-making into daily clinical practice is still a challenge for CMM pharmacists as it is for other health professionals, such as physicians seeking changes in their practices. Towle et al. [42] identified that professionals do not always offer alternatives to patients and that options are rarely provided fully, coherently and unbiased. After a systematic review of the literature, Couet and colleagues [44] found that the extent to which health professionals involve patients in decision-making is generally low. Even though pharmacists in this study highlight their efforts to share their decisions with patients, it is still a timid initiative because neither professionals nor patients are prepared to engage with this initiative. Furthermore, patients need the knowledge and power to engage in this process [45].

The theoretical framework of pharmaceutical care creates a promising ground for a joint decision between patient and pharmacist. However, in practice, patient participation has been stimulated at different levels by pharmacists, as these results suggest. Interventions to enhance the ability of pharmacists to involve patients at a maximum level, which enables shared decision-making, may be necessary.

#### 3.2.4. Factors That Can Modify the Decision-Making Process

The understanding of the decision-making process of the pharmacist during the provision of CMM revealed some factors that interfere with and are able to modify this process. They may entail a change or an adaptation of the decision. Among these factors is the professional’s interpretation of the meaning of considering the patient as a whole, professional autonomy, the absence of the patient at the time of the decision, pharmacist’s technical knowledge, and the context of the patient and professional. Professionals need to be aware of these factors so that they do not compromise the quality of the provided care, depriving patients of achieving the best possible results from CMM. In the presented model, these factors appear on dotted lines that depart or pass through the steps of the decision-making process that can be modified by the respective factor.

The philosophy of pharmaceutical care determines that the patient be cared for holistically, without fragmentation [46]. The participants of this study demonstrated that understanding the patient as a whole is an important part of their decision-making process. However, a variety of perspectives and definitions were observed regarding the meaning of considering the person holistically. The narrative below shows the anguish of the professional when having to solve other patient problems not directly associated with their pharmacotherapy:

“*I feel like an octopus with multiple arms. So it turns out that I make a decision about pharmacotherapy, I make a decision about non-pharmacological measures, I make a decision about other areas… because the patient is not just the medication he takes; he is a whole. Thus, to meet this need of the whole, of the complex being that he is, I must make other decisions…*”

*P2*

For the patient’s benefit, pharmacists end up extrapolating the scope of their practice. The interpretation that considering the patient holistically means meeting all of his/her demands can divert the pharmacist from her primary focus on patient care: identifying and solving drug-therapy problems. The pharmacist should consider the patient’s complexity and all of his/her subjectivity to assist him/her in solving these problems in the best possible way, in a way that is contextualized to his/her life.

Another factor that may interfere with the pharmacist’s decision-making process is his/her autonomy during CMM service. For the fulfillment of his/her mission in this service, the pharmacist’s working tool is the medication, whose prescription is traditionally linked to medicine. Given this scenario, the pharmacist performs a decision-making process to reach the patient’s DTP and seek the solution, which is independent of the prescriber. However, often, in order to effectively implement the necessary changes in patients’ pharmacotherapy, the pharmacist will need to seek the physician.

Amidst the decisions successfully implemented, all participating pharmacists encountered this obstacle at some point:

“*So many times I came across situations where everything was ready, and the doctor just did not agree, and the patient needed it.*”

*P3*

The dialogue among professionals may be limited by the fact that the final decision on whether or not to change the prescription is to be taken solely by the physician. As discussed by Weiss and Sutton [47], prescribing has always been indicative of physicians’ clinical autonomy and professional power in society.

However, an interesting aspect of the pharmacist’s lack of autonomy over a prescription is precisely the need for discussion with the prescriber. Considering the role of medicine in the prescription, most of the time, it will be necessary to establish communication between the pharmacist and prescriber so that the problem can be solved. Thus, the decision does not come from a single professional. The responsibilities need to be shared to some degree. Even in the face of challenges, there is the opportunity to foster teamwork, and that is where pharmacists must go.

The pharmacist’s decision-making process, in general, is not concluded during patient care. After this step, the pharmacist may need to focus on the case to clarify any doubts, as well as establish partnerships with prescribers. Faced with the need to discuss with the physician to make changes in the patient’s pharmacotherapy, sometimes the pharmacists choose not to reveal all the information to the patient until the conversation with other professional has occurred:

“*… I have no habit of talking to the patient before discussing with the doctor, or if I depend on any other professional because I think this will further stress our relationship.*”

*P3*

The intention is to maintain the harmony of the pharmacist–patient–physician triad, preserving relationships. However, when deciding not to present the options to solve a problem to the patient because the pharmacist considers he/she must first discuss them with the physician, the professional ends up leaving the main interested party out of the process. As pharmacists and physicians discuss and make a decision, the patient does not participate. Although they can consider the patient’s medication experience in this process, this attitude prevents the possibility of sharing the decision with the served person. Schafer, Gionfriddo and Boehm [48] also emphasize in their study that the need to confirm the decision with the physician harms the SDM process during CMM.

Notably, the technical knowledge of the professional can change their decision-making process. As one of the participants emphasizes, solid knowledge in pharmacotherapy favors problem identification as well as facilitates the sharing of the decision, as the professional will have subsidies to present options to the patient:

“*If you do not have a solid foundation in pharmacology, first you do not identify the problem, and second you cannot draw up different solutions… when you have a more solid knowledge of pharmacotherapy, you can even think of different solutions to present them to the individual.*”

*P11*

In accordance with that pointed out by the pharmacist above and with the results of this paper, Ribeiro and Amaral [49] highlight how important it is for the professional to have the security of his/her knowledge to discuss decisions with the patient, allowing for questioning. According to these authors, a critical updating of scientific knowledge is necessary to occur the sharing of the decision with the patient [49].

The context of the insertion of the professional also influences the decision-making process. The different trajectories of the pharmacists of this study with the CMM service and the experience of some of them with distinct practice scenarios allowed the understanding of variations produced in this process. Considering the aspects already described in this paper about the importance of a partnership between pharmacist and prescriber, one of the participants details how being in a scenario with other professionals favors their practice:

“*There is a case that happened recently, a patient who is an alcoholic and already has ascites due to his cirrhosis and I went to discuss with the doctor the treatment. He was using propranolol, which… worsens the survival of a patient who has ascites. When I went to discuss the case, she said, ‘no, propranolol is a choice,’ and I said, ‘It is not, let us open the algorithm, let us look.’ Then, the moment we opened it, she saw that any beta-blocker, any that decreases peripheral vascular resistance, could increase ascites, and then she changed it. If it were a letter, I would not have the chance to argue, and that happened too much.*”

*P2*

This narrative shows how the possibility of meeting the professionals and discussing the case leads to a more effective resolution of the patient problem. The indirect contact between pharmacist and prescriber can compromise the argument, making communication and, consequently, solving the patient problem difficult.

Pharmacist P4 provides CMM services in a scenario where there is no multi-professional team. Even with the proposal of working in collaboration with the prescribers, the geographical barrier ends up compromising this contact. In fact, not sharing the same location is pointed out as one of the difficulties for the development of collaborative practice, mainly due to limited casual and informal interactions [50]. In her narrative, this participant points out other challenges in this context, such as limited information:

“*In many situations, it is more difficult because, sometimes, I do not have a patient history. I have what the patient tells me. I often have tests that can corroborate what the patient told me, but I only have the story told by the patient. I do not know the patient’s health team.*”

*P4*

Faced with this scenario, where pharmacists may not have access to all the information they deem necessary, they need to evaluate all possibilities/hypotheses and make decisions with what they have available. In addition, in this context of the absence of contact with other health professionals, the role of the patient is emphasized. The patient is a link between professionals, and he/she will always participate in some degree of decisions.

On the other hand, inserted in the health team, the professional that performs CMM has the opportunity to gain the confidence of other professionals, showing how they can work collaboratively. Pharmacists, after consolidating their role with the team, begin to intervene directly with the patient regarding pharmacotherapy, with the support of the prescriber:

“*… He [the doctor] chooses a patient that he cannot adjust his dose of warfarin and directs him to me, that’s fantastic.*”

*P3*

The characteristics of each context can have positive or negative impacts on the pharmacist’s decision-making process. As participants demonstrated, it is necessary to take advantage of the benefits created by the context and try to overcome the barriers found so that these nuances do not affect the quality of the care provided to the patient and compromise the clinical results of these patients.

### 3.3. The Proposed Theoretical Model

Figure 1 shows the theoretical structure representative of the decision-making process during the delivery of CMM services by the participants in this study. The pharmacist’s decision-making process starts by associating pharmacotherapeutic knowledge with the patient’s medication experience as she/he collects the information to evaluate the indication, effectiveness, safety, and convenience of the drug-therapy in use by the patient. After identifying the problem, during the definition of the plan, these different types of knowledge are employed once again. At this stage, the patient is involved in various degrees. In the most desirable degree of involvement, the pharmacist and the patient come to a shared decision. The search for a holistic view of the patient runs through this whole process.

Through the stages of the decision-making process, the participants uncovered several factors that influence and can modify this process, such as the professional’s interpretation when considering the patient as a whole; professional autonomy; absence of the patient at the time the decision is being made; the pharmacist’s technical knowledge and the context of the patient and the environment in which the pharmacist practices. The emergent model illustrates the relationship of these factors with other steps that compose the decision-making process when taking care of patients in CMM services. Because these factors are not permanent components of the process, dashed lines were used to represent them in the theoretical structure.

In addition, the developed model does not reveal the consequences of the decision-making process or how new decisions are made as the results of previous ones. Certainly, this proposal is not conclusive. However, it hopes to make explicit what was initially implicit or in the head of pharmacists, helping them to become more aware of their decision-making process so that they can reflect on it, improve upon it and teach it.

## 4. Conclusions

The broad understanding of the pharmacist’s decision-making process allowed the construction of a theoretical proposal that explains this process. It is hoped that the knowledge generated in this study can be incorporated into the daily practice of pharmacists providing CMM services. It is also expected that it be applied as a basis for the development of educational interventions for the training of professionals competent to make decisions. Although the results of this study came from a limited number of Brazilian pharmacists, the results can be transferred and applied to pharmacists from other places who are engaged in the care for patients using pharmaceutical care practice as the foundation of CMM services.

## Figures and Tables

**Table 1:** Description of participants.

Participating Pharmacists	Setting Providing CMM Services	Period of Time in Practice (Years) *	Number of Patients Assisted *
P1	Primary care clinic	10	More than 150
P2	Specialty clinic	8	More than 400
P3	Primary care clinic	2	200
P4	Public Pharmacy	1	50
P5	Clinic at university	13	More than 500
P6	Primary care clinic	3	69
P7	Primary care clinic	1	65
P8	Specialty clinic	2	More than 200
P9	Specialty clinic	1	20
P10	Primary care clinic	1	200
P11	Clinic at university	16	More than 300

* Up to date of the interview.

## Appendix A. Interview Guide

**Topic 1**—*To capture the pharmacist’s perception of her practice.*

I would like you to tell me a little about your experience with the provision of CMM services. How has that experience been for you?

**Topic 2**—*To understand the decision-making process from the perspective of professional (what decisions she/he believe should be made during the offering of CMM and what it is like for him or her).*

As I told you, I am interested in the process you use to make decisions during the patient care process in the CMM service.

When I say decision-making, what does that mean to you?

In your opinion, how do you make decisions? What do you take into consideration? How do you think? Do you often reflect on your decision-making process during the provision of CMM? Tell me more about it. Could you give me one example of how this happens in practice?

What decisions do you think you should make during the CMM?

How do you feel when making a decision?

**Topic 3**—*To understand how the professional directs his consultations.*

When you are with the patient, what information do you consider crucial for your decision making? Please give me an example.

How does the patient participate in your process of making decisions? Which role does your patients take in this process?

**Topic 4**—*To analyze whether the rational decision-making process is incorporated by the pharmacist.*

How do you assess the medications in use by a patient? Do you use any specific reasoning? Tell me about it.

Do you think there has been an evolution in your thinking process? Can you tell me step by step how you got to a certain decision?

**Topic 5**—*To understand how the patient’s subjective experience with medications is considered in the pharmacist’s decision-making.*

Tell me a little about the patient’s experience with the use of medicines. What is the role of these experiences in your practice? How do you use this information? Please give me an example.

**Topic 5**—*To explore the skills and knowledge needed to make decisions.*

In your opinion, what should a pharmacist have or know to be more effective in making good decisions? Could you give me an example from your practice?

**Final question:** *Is there anything else about how you make your decisions you would like to comment on?*

## References

[1] Patient-Centered Primary Care Collaborative The Patient-Centered Medical Home: Integrating Comprehensive Medication Management to Optimize Patient Outcomes. June 2012. https://www.pcpcc.org/sites/default/files/media/medmanagement.pdf.

[2] Cipolle R.J., Strand L.M., Morley P.C. (2012). Pharmaceutical Care Practice: The Patient-Centered Approach to Medication.

[3] Neves C.M., Nascimento M.M.G.D., Silva D.Á.M., Ramalho-De-Oliveira D. (2019). Clinical Results of Comprehensive Medication Management Services in Primary Care in Belo Horizonte. Pharmacy.

[4] Budlong H., Brummel A., Rhodes A., Nici H. (2018). Impact of Comprehensive Medication Management on Hospital Readmission Rates. Popul. Health Manag..

[5] Mendonça S.A.M., Melo A.C., Pereira G.C.C., Santos D.M.D.S.D., Grossi E.B., Sousa M.D.C.V.B., Ramalho-De Oliveira D., Soares A.C. (2016). Clinical outcomes of medication therapy management services in primary health care. Braz. J. Pharm. Sci..

[6] Detoni K.B., Oliveira I.V., Nascimento M.M., Caux T.R., Alves M.R., Ramalho-De-Oliveira D. (2016). Impact of a medication therapy management service on the clinical status of patients with chronic obstructive pulmonary disease. Int. J. Clin. Pharm..

[7] Ramalho-De Oliveira D., Brummel A.R., Miller D.B. (2010). Medication Therapy Management: 10 Years of Experience in a Large Integrated Health Care System. J. Manag. Care Pharm..

[8] Obreli-neto P.R., Marusic S., Guidoni C.M., Baldoni A.O., Renovato R.D., Pilger D., Cuman R.K., Pereira L.R. (2005). Economic evaluation of a pharmaceutical care program for elderly diabetic and hypertensive patients in primary health care: A 36-moa 36-month randomized controlled clinical trial. J. Manag. Care Spec. Pharm..

[9] Patient Care Process for Delivering Comprehensive Medication Management (CMM): Optimizing Medication Use in Patient-Centered, Team-Based Care Settings CMM in Primary Care Research Team. July 2018. http://www.accp.com/cmm_care_process.

[10] Freitas E.L., Ramalho-De-Oliveira D. (2015). Critical thinking in the context of clinical practice: The need to reinvent pharmacy education. Rev. Port. Educ..

[11] Bartels C.E. (2013). Analysis of Experienced Pharmacist Clinical Decision-Making for Drug Therapy Management in the Ambulatory Care Setting. Ph.D. Thesis.

[12] LaDuca A., Engel J.D., Chovan J.D. (1988). An Exploratory Study of Physicians’ Clinical Judgment. Eval. Health Prof..

[13] Burman M.E., Stepans M.B., Jansa N., Steiner S. (2002). How do NPs make clinical decisions?. Nurse Pract..

[14] Hedberg B., Larsson U.S. (2003). Observations, confirmations and strategies—Useful tools in decision-making process for nurses in practice?. J. Clin. Nurs..

[15] Hoffman K., Donoghue J., Duffield C. (2003). Decision-making in clinical nursing: Investigating contributing factors. J. Adv. Nurs..

[16] Mamede S., Schmidt H.G., Rikers R.M., Penaforte J.C., Coelho-Filho J.M. (2007). Breaking down automaticity: Case ambiguity and the shift to reflective approaches in clinical reasoning. Med. Educ..

[17] Traynor M., Boland M., Buus N. (2010). Autonomy, evidence and intuition: Nurses and decision-making. J. Adv. Nurs..

[18] Dickson G.L., Flynn L. (2011). Nurses’ Clinical Reasoning: Processes and practices of medication safety. Qual. Health Res..

[19] Marcum J.A. (2013). The Role of Emotions in Clinical Reasoning and Decision Making. J. Med. Philos..

[20] Wainwright S.F., Shepard K.F., Harman L.B., Stephens J. (2011). Factors That Influence the Clinical Decision Making of Novice and Experienced Physical Therapists. Phys. Ther..

[21] Simmons B. (2010). Clinical reasoning: Concept analysis. J. Adv. Nurs..

[22] Simmons B., Lanuza D., Fonteyn M., Hicks F., Holm K. (2003). Clinical Reasoning in Experienced Nurses. West. J. Nurs. Res..

[23] McBane S.E., Dopp A.L., Abe A., Benavides S., Chester E.A., Dixon D.L., Dunn M., Johnson M.D., Nigro S.J., American College of Clinical Pharmacy (2015). Collaborative Drug Therapy Management and Comprehensive Medication Management―2015. Pharmacother. J. Hum. Pharmacol. Drug Ther..

[24] Butler A., Dehner M., Gates R.J., Shane P.A., Chu M., DeMartini L., Stebbins M., De Ybarra J.N., Peck C., McInnis T. (2017). Comprehensive Medication Management programs: 2015 status in Southern California. Res. Soc. Adm. Pharm..

[25] DiPiro J.T., Talbert R.L., Yee G.C., Matzke G.R., Wells G.B., Posey M. (2016). Pharmacotherapy: A Pathophysiologic Approach.

[26] Mendonça S.A.M., Meireles B.L., Freitas E.L., Ramalho-De Oliveira D. (2017). Pharmacy Practice Experiential Programs in the Context of Clinical Education. Int. J. Pharm. Pharm. Sci..

[27] Charmaz K. (2009). A Construção da Teoria Fundamentada: Guia Prático para Análise Qualitative.

[28] Daly K.J. (2007). Qualitative Methods for Family Studies and Human Development.

[29] Strauss A., Corbin J. (2008). Basics of Qualitative Research: Techniques and Procedures for Developing Grounded Theory.

[30] Ramalho-de-Oliveira D. (2010). The Reality of Pharmaceutical Care Based Medication Therapy Management: Patients’, Pharmacists’ and Students’ Perspectives.

[31] Freitas E.L. (2014). Why Do I Think the Way I do? Troubling the Concept of Critical Thinking in Pharmacy Classrooms. Ph.D. Thesis.

[32] Facione N.C., Facione P.A. (2008). Critical thinking and clinical judgment. Critical Thinking and Clinical Reasoning in the Health Sciences: A Teaching Anthology.

[33] Sorensen T.D., Hager K.D., Schlichte A., Janke K. (2020). A Dentist, Pilot, and Pastry Chef Walk into a Bar… Why Teaching PPCP is Not Enough. Am. J. Pharm. Educ..

[34] Losinski V. (2011). Educating for Action: Understanding the Development of Pharmaceutical Care Practitioners. Ph.D. Thesis.

[35] Aleluia I.M.B., Carvalho P.M., Menezes M.S. (2010). A way to assess students’ clinical reasoning. Med. Educ..

[36] Etminan M., Wright J.M., Carleton B. (1998). Evidence-based pharmacotherapy: Review of basic concepts and applications in clinical practice. Ann. Pharmacother..

[37] Adams R. (2009). Health literacy—A new concept for general practice?. Aust. Fam. Phys..

[38] Elwyn G., Frosch D.L., Thomson R., Joseph-Williams N., Lloyd A., Kinnersley P., Cording E., Tomson D., Dodd C., Rollnick S. (2012). Shared Decision Making: A Model for Clinical Practice. J. Gen. Intern. Med..

[39] Elwyn G., Dehlendorf C., Epstein R.M., Marrin K., White J., Frosch D.L. (2014). Shared Decision Making and Motivational Interviewing: Achieving Patient-Centered Care across the Spectrum of Health Care Problems. Ann. Fam. Med..

[40] Freitas E.L. (2005). Revelando a Experiência do Paciente com a Prática da Atenção Farmacêutica: Uma Abordagem Qualitativa. Master’s Thesis.

[41] De Caux T.R. (2015). Meus Filhos me Perguntam, ‘por que Consulta com a Farmacêutica?’ Experiência de Pacientes com um Serviço de Gerenciamento da Terapia Medicamentosa. Undergraduate Thesis.

[42] Towle A., Godolphin W., Grams G., Lamarre A. (2006). Putting informed and shared decision making into practice. Health Expect..

[43] Gwyn R., Elwyn G. (1999). When is a shared decision not (quite) a shared decision? Negotiating preferences in a general practice encounter. Soc. Sci. Med..

[44] Couët N., Desroches S., Robitaille H., Vaillancourt H., Leblanc A., Turcotte S., Elwyn G., Légaré F. (2015). Assessments of the extent to which health-care providers involve patients in decision making: A systematic review of studies using the OPTION instrument. Health Expect..

[45] Joseph-Williams N., Elwyn G., Edwards A. (2014). Knowledge is not power for patients: A systematic review and thematic synthesis of patient-reported barriers and facilitators to shared decision making. Patient Educ. Couns..

[46] Ramalho-de-Oliveira D. (2011). Atenção Farmacêutica: Da Filosofia ao Gerenciamento da Terapia Medicamentosa.

[47] Weiss M.C., Sutton J. (2009). The changing nature of prescribing: Pharmacists as prescribers and challenges to medical dominance. Sociol. Health Illn..

[48] Schafer K.M., Gionfriddo M.R., Boehm D.H. (2016). Shared decision making and medication therapy management with the use of an interactive template. J. Am. Pharm. Assoc..

[49] Ribeiro M.M.F., Amaral C.F.S. (2008). Medicina centrada no paciente e ensino médico: A importância do cuidado com a pessoa e o poder médico. Rev. Bras. Educ. Méd..

[50] Legault F., Humbert J., Amos S., Hogg W., Ward N., Dahrouge S., Ziebell L. (2012). Difficulties Encountered in Collaborative Care: Logistics Trumps Desire. J. Am. Board Fam. Med..

